# Supplementary figures and images for: A tunable affinity fusion tag for protein self-assembly
Source: bioRxiv. 2025 Jan 15:2025.01.14.633037. Preprint. [Version 1] doi: 10.1101/2025.01.14.633037 (PMC11761134; doi:10.1101/2025.01.14.633037)

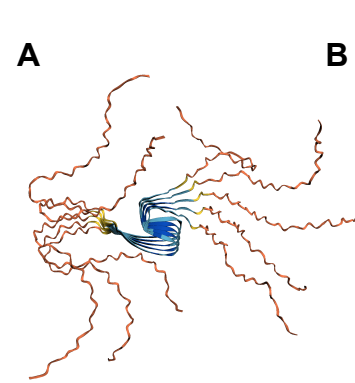

**B**

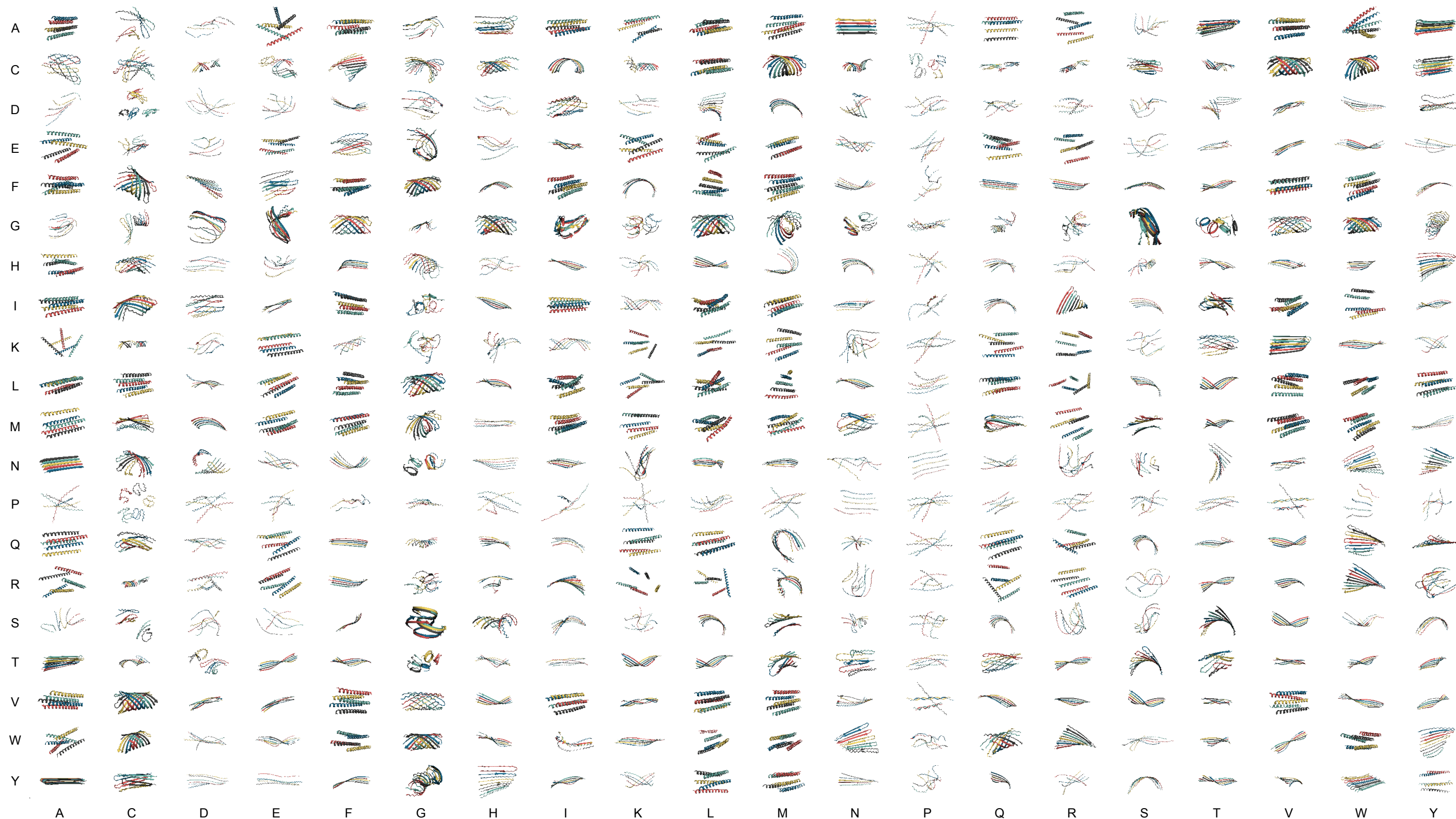

Supplement: Supplement 1 — Figure S1. Additional data supporting main figures. (a) AlphaFold structural prediction of human functional amyloid Ripk3 (413-512). (b) AlphaFold pentamer predictions of all dipeptide repeat combinations at total peptide length 40 amino acids, colored by chain. (c). SDD-AGE gel prepared from [pin-] yeast cells expressing (TA)20, the intrinsically disordered domain of TDP43, and an amyloid forming polyQ tract where the dashed line represents the two adjacent groups of lanes are spliced from different positions in the gel. (d) Representative confocal microscopy image of yeast cells expressing (TTAA)10 at a range of intracellular concentrations. Scale bar, 10 μm. (e) AlphaFold structural predictions of (TA)20 (left) and (TTAA)10 (right) where backbone is colored by pLDDT and side chains are colored green (alanine) and grey (threonine). [file media-1.pdf]
